# Two novel mutations of TACSTD2 found in three Japanese gelatinous drop-like corneal dystrophy families with their aberrant subcellular localization

**Published:** 2011-04-19

**Authors:** Mina Nakatsukasa, Satoshi Kawasaki, Kenta Yamasaki, Hideki Fukuoka, Akira Matsuda, Kohji Nishida, Shigeru Kinoshita

**Affiliations:** 1 Department of Ophthalmology, Kyoto Prefectural University of Medicine, Kyoto, Japan; 2 Department of Ophthalmology, Juntendo University School of Medicine, Tokyo, Japan; 3 Department of Ophthalmology, Osaka University Graduate School of Medicine, Osaka, Japan

## Abstract

**Purpose:**

To report two novel mutation of the tumor-associated calcium signal transducer 2 (*TACSTD2*) gene in 3 Japanese patients with gelatinous drop-like corneal dystrophy (GDLD).

**Methods:**

Genomic DNAs were extracted from the peripheral blood of 3 Japanese families. The coding region of *TACSTD2* was amplified by polymerase chain reaction (PCR) and subjected to direct sequencing analysis. Plasmid vectors harboring normal and mutated *TACSTD2* were transfected to the immortalized human corneal epithelial cells to identify the subcellular localization of the normal and mutated *TACSTD2* gene products.

**Results:**

Sequencing analysis of *TACSTD2* revealed two novel homozygous mutations (c.840_841insTCATCATCGCCGGCCTCATC and c.675C>A which may result in frameshift (p.Ile281SerfsX23) and nonsense (p.Tyr225X) mutations, respectively) in the 3 GDLD patients. Protein expression analysis showed that the mutated gene product was distributed diffusely in the cytoplasm, whereas the normal gene product accumulated at the cell-to-cell borders.

**Conclusions:**

This study reports two novel mutations in 3 GDLD families and expands the spectrum of mutations in *TACSTD2* that may cause pathological corneal amyloidosis.

## Introduction

Gelatinous drop-like corneal dystrophy (GDLD; OMIM 204870) was first described by Nakaizumi [1] as an uncommon, autosomal recessive disease, characterized by bilateral corneal amyloidosis. To date, this disease is still quite rare in many countries, however, it is relatively common in Japan with a prevalence rate of 1 in 31,546 individuals as estimated from the frequency of parental consanguinity [2,3]. In the first decade of the lives of GDLD patients, grayish, subepithelial nodular amyloid depositions appear and result in severe photophobia, lacrimation, and an ocular foreign body sensation [4,5]. As the patients age, the amyloid depositions typically enlarge, increase in number, coalesce, and exhibit a mulberry-like appearance, thus leading to severe bilateral vision loss usually beginning within the third decade of the patients’ lives.

Tsujikawa et al. [6] revealed through the use of a linkage analysis and consecutive candidate gene approach that the specific gene responsible for this disease is tumor-associated calcium signal transducer 2 (*TACSTD2*). To date, fifteen reports have demonstrated twenty-three different GDLD-causing alterations in *TACSTD2* comprised of nine missense-, five nonsense-, and nine frameshift-causing (deletion and insertion) mutations from nine different geographical regions including Japan, China, India, Iran, Tunisia, Estonia, Turkey, Vietnam, and Europe, most of which used to be developing regions with a predominance of consanguineous marriage [6-15]. In the present study, we report two novel *TACSTD2* mutations from 3 Japanese GDLD patients.

## Methods

### Ethical issues

All experimental procedures were approved by the Institutional Review Board for Human Studies at Kyoto Prefectural University of Medicine, Kyoto, Japan. Prior informed consent was obtained from all patients after a detailed explanation of the study protocols, and this study was performed in accordance with the tenets of the Declaration of Helsinki for research involving human subjects.

### Subjects

All patients were given a complete ophthalmic examination including visual acuity testing, noncontact tonometry, and slit-lamp examination. For all 3 GDLD patients enrolled in this study, clinical diagnosis was confirmed based upon slit-lamp examination and the agreement of at least 2 corneal specialists in our department.

### Sequencing analysis

Genomic DNA was extracted from peripheral blood using a commercially available column-based DNA extraction kit (DNeasy^®^ Blood & Tissue Kit; QIAGEN GmbH, Hilden, Germany). Sequencing analysis was performed using a commercially available kit (BigDye 3.1; Applied Biosystems, Inc., Foster City, CA). Polymerase chain reaction (PCR) was performed with a primer pair against *TACSTD2* (M1S1-F-2; 5′-CCT GCA GAC CAT CCC AGA C-3′, M1S1-R-2; 5′-CAG GAA GCG TGA CTC ACT TG-3′) which fully covered the coding sequence of this gene. The PCR product was bi-directionally sequenced in a 20-μl reaction buffer containing a 2× sequencing mixture and either of the above primers. After ethanol precipitation, the sequence products were electrophoresed on an automated capillary sequencer (Genetic Analyzer 3130xl; Applied Biosystems).

### Validation of the sequencing data

As for the family members related to Case 1 and Case 2, sequencing data was validated by PCR using a primer pair (M1S1–20ins-F; 5′-TGA AGC GCC TCA CCG CCG GC-3′, M1S1–20ins-R; 5′-CGA CGA GGG CCA CCA CGA CC-3′) which encompass the site of the identified insertional mutation.

As for Case 3, sequencing data was validated by the single-base primer extension assay with a commercially available kit (SNaPshot^®^ Multiplex System; Applied Biosystems) with a primer (SS-M1S1-Y225X: 5′-ATC GGC GAT GCC GCC TAC TA-3′).

### Plasmid construction

For the protein expression of either the wild-type or mutated *TACSTD2*, DNA fragments covering an entire open reading frame with or without particular mutations were amplified by PCR, ligated into an expression vector pcDNA3.1/V5-His-TOPO (Invitrogen Corp., Carlsbad, CA), and transformed into chemically competent cells (JM109; TOYOBO Co., Ltd., Osaka, Japan). A single colony, which was confirmed via sequencing analysis to have the proper expected sequence without any unexpected mutations, was isolated, propagated, and subjected to the plasmid extraction using a commercially available column-based kit (NucleoBond; MACHEREY-NAGEL GmbH & Co., Düren, Germany).

### Cell culture and gene transfer

SV40 immortalized human corneal epithelial (HCE-T) cells [16] were subcultured every 4 days and maintained in DMEM/F12 containing 200 U/ml penicillin and streptomycin, 10% fetal bovine serum (FBS; Cellgro; Mediatech, Inc., Herndon, VA), 0.1 μg/ml cholera toxin (List Biologic Laboratories, Inc., Campbell, CA), 5 μg/ml insulin (Sigma-Aldrich Corp., St. Louis, MO), and 10 ng/ml human epidermal growth factor (Invitrogen). After the cells had reached to 70%–80% confluency on a commercially available culture-glass slide (Nunc Lab-Tek™ Chamber Slide™ System; Thermo Fisher Scientific, Inc., Rochester, NY), each of the plasmids was transfected into the HCE-T cells using Lipofectamine™ LTX (Invitrogen) according to the manufacturer’s instructions.

### Immunocytostaining analysis

Cells grown on the culture-glass slide were fixed with Zamboni’s fixative, blocked with 1% skim milk, and then incubated overnight with a primary antibody at 4 °C. The primary antibody included anti-V5 (MM IgG_1_, clone V5005; NACALAI TESQUE, Inc., Kyoto, Japan) and normal mouse IgG_1_ (Dako Denmark A/S, Glostrup, Denmark) as a negative control. After being washed with 0.01 M of phosphate buffered saline (PBS), the samples were incubated with a secondary antibody (Alexa Fluor® 488-labeled anti-mouse or anti-goat IgG; Invitrogen) at room temperature for 1 h. After being washed again with 0.01 M PBS, the sections or the cells were counter-stained with propidium iodide, mounted, covered with coverslips, and observed and photographed using a fluorescence microscope (AX70 TRF; Olympus Corporation, Tokyo, Japan).

## Results

### Clinical findings

**Figure 1:**
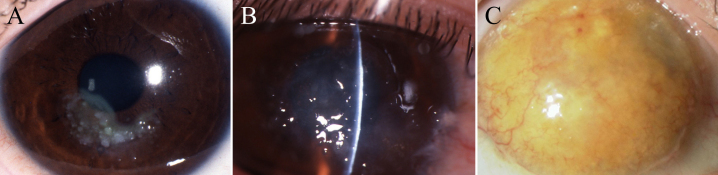
Images demonstrating the corneas of 3 unrelated GDLD patients. Proband A (**A**) and proband B (**B**) demonstrated mulberry-type GDLD corneas with multiple grayish subepithelial amyloid depositions. Proband C (**C**) demonstrated a kumquat-like GDLD cornea with neovascularization.

Case 1 involved a 30-year-old Japanese man (proband A) who had undergone photorefractive keratectomy (PTK) in his right eye at the age of 16 and in his left eye at the age of 21. His parents were second cousins to one-another. He had bilateral diffuse corneal opacities with multiple grayish-white nodular elevations located at the subepithelial region (Figure 1A) which fit the classification of typical mulberry GDLD [17].

Case 2 involved a 29-year-old Japanese female (proband B) who had undergone PTK in her left eye at the age of 23 and in her right eye at the age of 26. Her parents were first cousins to one-another. Slit-lamp examination revealed grayish amyloid depositions in the bilateral corneas which fit the classification of typical mulberry GDLD (Figure 1B).

Case 3 involved an 83-year-old Japanese woman (proband C). Her parents’ marriage was not consanguineous. She had undergone lamellar keratoplasty along with keratoepithelioplasty in her left eye at the age of 72 and penetrating keratoplasty in her right eye at the age of 82. Slit-lamp examination revealed the characteristic findings of a kumquat-like GDLD subtype with neovascularization in both of her eyes (Figure 1C). Recurrence of amyloid deposition was observed in both of her eyes. Surface keratectomy was performed for her left eye to remove the superficial amyloid depositions, but no surgical intervention was undertaken for her right eye because she was too elderly to undergo the operation at that time.

All surgeries for the 3 cases were performed to treat their GDLD corneas. After the surgeries, case 1 and case 2 continued to wear soft contact lenses and no recurrence was observed in the eyes of those patients, however, case 3 was unable to wear soft contact lenses and recurrence occurred in both of her eyes. Those findings are in good agreement with the previous study that reported the protective effect of using a soft contact lens for the postoperative GDLD cornea [18].

### Mutation analysis

**Figure 2:**
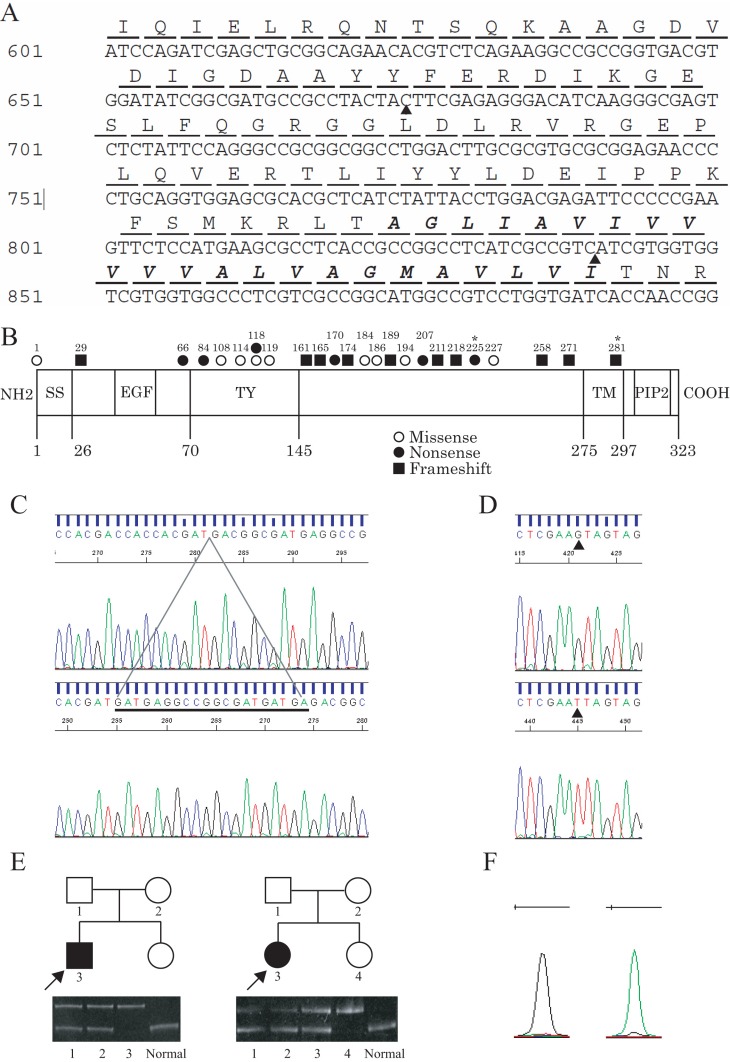
Results of sequencing analysis, PCR analysis, and single-base primer extension assay. **A**: Nucleotide and amino acid sequence of TACSTD2. Arrowheads indicate the site of the c.675C>A and c.840_841insTCATCATCGCCGGCCTCATC nucleotide changes. Note that the amino acids in bold italic type are of the transmembrane domain. **B**: Computationally-predicted domain structure of the TACSTD2 protein with mutations of previous reports and this report (*). SS: signal sequence; EGF: EGF-like repeat; TY: thyroglobulin type I repeat; TM: transmembrane domain; PIP2: PIP2 binding sequence. **C**: Results of sequencing analysis of *TACSTD2* in a normal volunteer (upper) and in proband A or B (lower). The underlined nucleotides indicate the inserted 20-base sequence between the 840th and 841st nucleotide positions of *TACSTD2*. Note that the presented sequence is in a reverse direction. **D**: Results of sequencing analysis of *TACSTD2* in a normal volunteer (upper) and in proband C (lower). Arrowheads indicate the site of the c.675C>A mutation. Note that the presented sequence is in a reverse direction. **E**: Results of PCR analysis to examine the difference in length between the normal and insertion-bearing alleles in the families of the proband A (left) and proband B (right). The upper bands indicate the PCR product derived from the insertion-bearing alleles while the lower bands indicate the PCR product from the normal alleles. Note that the sister of proband A was not examined. **F**: Results of 1-base primer extension analysis for the 675th nucleotide of *TACSTD2* in the normal volunteer (left) and the proband C (right). Black indicates C and green indicates A. Note that the presented data was produced by the forward primer.

Sequencing analysis of *TACSTD2* revealed a homozygous, 20-base insertion mutation between the 840th and the 841st nucleotide positions (c.840_841insTCATCATCGCCGGCCTCATC) for proband A and proband B (Figure 2C), resulting in a putative frameshift and a premature termination at the 303th amino acid position (p.Ile281SerfsX23). The respective parents of the proband A and proband B, as well as the younger sister of proband B, all of whom had no abnormal findings in their corneas, had one allele with a mutated *TACSTD2* gene and one allele with a wild-type *TACSTD*2 gene (Figure 2E), indicating that the phenotype well co-segregates with the genotype in these pedigrees. Proband C was found to have a homozygous substitutive mutation from C to A at the 675th nucleotide position (c.675C>A), which may result in nonsense mutation at the 225th amino acid position (p.Tyr225X; Figure 2D). The sequence data were further validated by the difference in the length of the PCR products for proband A, proband B, and their respective family members or by the single-base primer extension analysis for proband C (Figure 2). Data for other family members related to proband C were not obtained due to the fact they refused permission to be enrolled in this study.

### Subcellular localization of TACSTD2 protein

**Figure 3:**
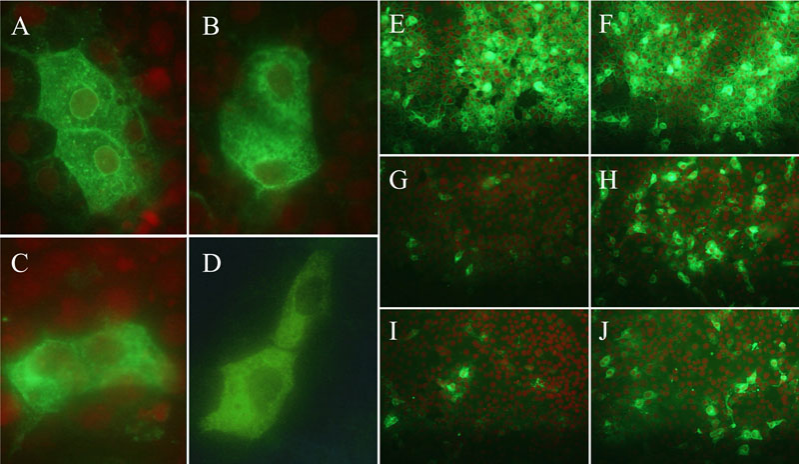
Results of the immunocytostaining analysis using anti-V5 antibody for the HCE-T cells transfected with expression vector harboring the wild-type or mutated TACSTD2 gene tagged with V5-epitope. Immunolocalization at the plasma membrane is apparent in the HCE-T cells transfected with the wild-type (**A**) *TACSTD2*. In HCE-T cells transfected with the mutated (**B**: p.Ile281SerfsX23, **C**: p.Tyr225X, **D**: p.Gln118X) *TACSTD2*, immunoreactivity was observed not at the plasma membrane but in the cytoplasm with slightly intensified signal around their nucleus. In the HCE-T cells transfected with wild-type *TACSTD2* (**E** and **F**), no apparent change was observed by the detergent treatment (0.1% Tween-20 for 30 min; **F**). However, in the HCE-T cells transfected with mutated *TACSTD2* (**G** and **H**: p.Ile281SerfsX23, **I** and **J**: p.Tyr225X), detergent treatment (**H**, **J**) significantly increased the number of the immunopositive cells as compared to those with no detergent treatment (**G** and **I**).

The V5-epitope tagged expression plasmid vector harboring either wild-type or mutated TACSTD2 protein was transfected into the HCE-T cells. Immunocytological staining analysis using anti-V5 antibody against the transfected HCE-T cells revealed that the normal TACSTD2 protein distributes both at the plasma membrane and in the cytoplasm while the mutated TACSTD2 protein was found to be diffusely distributed in the cytoplasm with no apparent plasma membrane localization (Figure 3). Detergent treatment with 0.1% Tween-20 significantly increased the number of V5-immunopositive cells among the cells transfected with the mutated *TACSTD2* gene, confirming the cytoplasmic localization of the mutated TACSTD2 protein.

## Discussion

In this study, we have identified two novel homozygous mutations from 3 unrelated GDLD patients with a phenotype well co-segregated with the genotype within their respective families. The insertional mutation of *TACSTD2* that was found in 2 of the GDLD patients may have resulted from a flame-shift amino acid alteration with premature termination (p.Ile281SerfsX23) within the transmembrane domain. A substitutive mutation found in 1 of the GDLD patients may have resulted from a nonsense mutation (p.Tyr225X) within a region between the thyroglobulin type-1 and transmembrane domains. The transmembrane domain should support the hydrophobic scaffold which may be fundamental to the membrane binding property of this protein. However, and as far as we know, such a domain structure is only a computationally speculated model from the primary amino acid structure of this protein. Therefore, the subcellular localization of both the wild-type and mutated TACSTD2 proteins was experimentally determined in this study.

Other than the changes in the subcellular localization of the TACSTD2 protein in the GDLD patients as identified in this current study, the functions of the TACSTD2 protein have yet to be elucidated. Using electron microscopy, Kinoshita et al. [19] demonstrated an enlarged intercellular space and facilitated scaling of the superficial cells of corneal epithelium in GDLD corneas. Quantock et al. [20] reported the increased permeability in the epithelium of the GDLD cornea using horseradish peroxidase as a molecular tracer. Takaoka et al. [21] found decreased expression of the tight junction–related protein including claudins (CLDNs), zonula occludens-1, and occludin in GDLD corneas. Recently, we discovered that the TACSTD2 protein directly binds to CLDN1 and 7 proteins and protects them from degradation by ubiquitin-proteasome system [22]. In the absence of functional TACSTD2 protein, the CLDN proteins will be degraded and tight junctions will not be formed, resulting in the hyperpermeation of tear fluid into the cornea, ultimately leading to the subepithelial deposition of amyloid in the cornea.

It has been reported that an AxxxG motif in the transmembrane domain of the epithelial cell adhesion molecule (EpCAM) protein, a paralogous gene of the *TACSTD2* gene, is involved in the binding of the EpCAM protein to the CLDN7 protein [23]. Since the transmembrane domain of the TACSTD2 protein also has the AxxxG motif at the corresponding site to the EpCAM protein [22], only the membrane-bound TACSTD2 protein seems to have the potential to execute the binding activity to CLDNs. Thus, we strongly believe that the mutated TACSTD2 protein being devoid of the binding property to the plasma membrane is actually pathological, as is shown in the present study.

Interestingly, the 20-base insertion mutation was found in 2 unrelated GDLD patients. Considering the fact that this mutation has thus-far not been reported, along with the fact that insertion mutations tend to be much rarer compared to substitution mutations, this mutation seems to be a founder mutation caused in a single Japanese ancestor, as has been reported in GDLD [6] and TGFBI-related corneal dystrophies [24-26]. Therefore, although these 2 GDLD patients are not related to one-another, they may have a common ancestor who may bear one de novo mutation of *TACSTD2*, possibly at one of his or her alleles.

In summary, we report here two novel mutations of *TACSTD2* and their altered subcellular localization in the corneal epithelial cells in vitro. The TACSTD2 protein may have various unidentified functions other than those that we have already shown, and we hope that the findings presented in this study will provide the next step toward a better understanding of the pathogenesis of GDLD.
